# In Silico Screening-Level Prioritization of 8468 Chemicals Produced in OECD Countries to Identify Potential Planetary Boundary Threats

**DOI:** 10.1007/s00128-017-2253-9

**Published:** 2017-12-28

**Authors:** Efstathios Reppas-Chrysovitsinos, Anna Sobek, Matthew MacLeod

**Affiliations:** Department of Environmental Science and Analytical Chemistry (ACES), Stockholm University, 10691 Stockholm, Sweden

**Keywords:** QSAR, Hazard screening, Planetary boundary, POPs, Persistence, Bioaccumulation, Long-range transport

## Abstract

Legislation such as the Stockholm Convention and REACH aim to identify and regulate the production and use of chemicals that qualify as persistent organic pollutants (POPs) and very persistent and very bioaccumulative (vPvB) chemicals, respectively. Recently, a series of studies on planetary boundary threats proposed seven chemical hazard profiles that are distinct from the POP and vPvB profiles. We previously defined two exposure-based hazard profiles; airborne persistent contaminants (APCs) and waterborne persistent contaminants (WPCs) that correspond to two profiles of chemicals that are planetary boundary threats. Here, we extend our method to screen a database of chemicals consisting of 8648 substances produced within the OECD countries. We propose a new scoring scheme to disentangle the POP, vPvB, APC and WPC profiles by focusing on the spatial range of exposure potential, discuss the relationship between high exposure hazard and elemental composition of chemicals, and identify chemicals with high exposure hazard potential.

**Electronic supplementary material:**

The online version of this article (10.1007/s00128-017-2253-9) contains supplementary material, which is available to authorized users.

The global production of synthetic chemicals is increasing exponentially by total volume (CEFIC 2014; ACC 2014) and in structural diversity (Binetti et al. 2008). The presence of synthetic chemicals in the environment is responsible for a plethora of effects that manifest themselves at scales from the molecular level up to the scale of earth system processes (Rockström et al. 2009; Steffen et al. 2015; Persson et al. 2013; Bernhardt et al. 2017; Diamond et al. 2015; MacLeod et al. 2014). To address the conceptual and data-related challenges associated with early identification of chemicals with unacceptable impacts, a number of databases (Boethling et al. 2004; Apodaca 2011), in silico physicochemical property estimation tools (Schüürmann et al. 2007; Sushko et al. 2011; Cao et al. 2008; Bennett et al. 2009) and models of chemical fate and transport (Arnot 2009) have been developed. Furthermore, a series of regulatory efforts that aim to identify chemicals of concern by screening against exposure and hazard criteria, and by defining profiles of contaminants of concern (European Parliament the Council of the European Union 2006; UNEP 2001) have been developed and deployed.

There is a growing number of computational studies in the scientific literature that aim to identify and prioritize chemicals that may have unacceptable environmental profiles (Walker and Carlsen 2002; Matthies et al. 2016; Strempel et al. 2012; Brown and Wania 2008; Muir and Howard 2006; Rorije et al. 2011; Scheringer et al. 2012). Among these studies, Screen-POP (Arnot et al. 2012; Breivik et al. 2012) focused on screening chemicals reportedly produced in the OECD countries to identify chemicals similar to persistent organic pollutants (POPs)—a chemical profile regulated under the Stockholm Convention—and persistent, bioaccumulative and toxic (PBT) substances, which are regulated under the European chemicals regulation, REACH. To this end, a database of 12,619 organic chemicals reportedly produced in the OECD countries was assembled from five national and international production volume lists, and a number of potential POP and PBT chemicals were identified (Arnot et al. 2012; Breivik et al. 2012). The Screen-POP project applied a “holistic” approach to quantitatively assess the exposure potential of the chemicals in the database, taking into account their physicochemical properties, their overall environmental partitioning and behavior, and their emissions. Moreover, uncertainties associated with emission and property estimates were also considered. A few chemicals identified in Screen-POP that were previously unknown as contaminants in the environment were later detected in the atmosphere and sediments near Stockholm, Sweden (McLachlan et al. 2014).

Recently, MacLeod et al. (2014) defined a set of chemical profiles for planetary boundary threats. The potential for chemicals to be planetary boundary threats is not considered in current regulatory regimes for PBT chemicals at local and regional scales, or for POPs at the global scale (Persson et al. 2013), and should therefore be considered independently in screening and prioritization exercises. We subsequently proposed a screening-level prioritization method to identify chemicals that fit two novel exposure-based profiles we derived from the planetary boundary threat framework (Reppas-Chrysovitsinos et al. 2017). These novel profiles assign high priority to persistent and mobile chemicals, which are labelled as airborne persistent contaminants (APCs) and/or waterborne persistent contaminants (WPCs). We applied an exposure-based screening method using our two proposed novel profiles, in parallel to profiles for POPs and very persistent and very bioaccumulative chemicals (vPvB), and evaluated the method using a set of 464 chemicals identified in an Arctic Monitoring and Assessment Programme (AMAP) report (AMAP 2017) as emerging Arctic contaminants of concern.

In this study, we apply the same methodology to 8648 chemicals taken from the Screen-POP database of 12,619 substances produced within OECD countries. Our goal is to apply our hazard scoring method to identify potential POP, vPvB, APC and WPC candidates among these chemicals. The Screen-POP database is larger than the AMAP database, which is composed only of known environmental contaminants, and it is also more structurally diverse. Therefore, the application of our method to the Screen-POP set of chemicals provides an opportunity to further investigate its prospective assessment capabilities, and to identify and prioritize chemicals that are POPs, vPvB, and potential planetary boundary threats from among those in the database.

## Materials and Methods

Our screening method is described in detail in our previous publication in which we screened known Arctic contaminants (Reppas-Chrysovitsinos et al. 2017). In brief, our method is based on deriving an exposure-based hazard scoring scheme for each of the POP, vPvB, APC and WPC profiles and using these scores to benchmark the case study chemicals as a percentile rank against a reference set of 148 chemicals with well-characterized environmental fate profiles. The four hazard scores are the product of selected combinations of hazard metrics for persistence (P), bioaccumulation (B) and long-range transport (LRT). The P and LRT metrics are calculated using the OECD Tool (Wegmann et al. 2009), a multimedia model that calculates overall persistence (*P*_ov_), and the three LRT metrics; transfer efficiency (*TE*), characteristic travel distance in air for emissions to air (*CTD*_air_) and characteristic travel distance in water for emissions to water (*CTD*_water_). Our B metric is calculated using EPI Suite’s (United States Environmental Protection Agency 2012) BCFBAF QSAR to obtain *BAF*, and thus considers both the hydrophobicity of the chemicals and their estimated potential for biotransformation.

Our exposure-based hazard profiles are calculated as the product of intensive or quasi-intensive chemical properties (P, B and LRT) that allow a meaningful comparison of chemicals (Mackay et al. 2001) for their potential to cause environmental or ecosystem exposure (Scheringer 2002). The profiles translate legislative language that uses the word “and” (e.g. “persistent and bioaccumulative”) as a logical AND operator and therefore we ascribed this as a multiplicative combination of the metrics.

The vPvB profile assigns high priority to very persistent and very bioaccumulative compounds without considering potential for long-range transport and is therefore a hazard profile that does not prioritize exposure due to long-range transport to regions remote from sources. The POP profile also includes multiplicative terms for persistence and potential for bioaccumulation, but also a term for long-range transport potential. Our LRT metric of choice for the POP profile is *TE* (%), which accounts for atmospheric transport and deposition in remote areas. Including deposition in the metric for LRT for the POP profile is appropriate since deposition is a prerequisite for exposure to aquatic biota, which is further enhanced by high potential for bioaccumulation. The choice of *TE* as LRT metric renders the POP profile of regional-to-hemispheric coverage. The APC and WPC profiles prioritize chemicals with hemispheric-to-global scale spatial range, as they include multiplicative terms for persistence and for mobility in air (*CTD*_air_, km) or water (*CTD*_water_, km).

We benchmarked the Screen-POP chemicals against a reference set of 148 contaminants for each of the four hazard profiles and assigned them scores (***S***_PROFILE_) as percentile ranks compared to the reference set. Most Screen-POP chemicals are thus assigned to a bin bounded by two reference set compounds that serve as the upper and lower limit of the bin. However, there are 149 bins per profile in total as the upper and lower bins are each bounded by one reference set chemical which is, respectively, the highest and the lowest scoring contaminant in this profile. Therefore, possible scores for the Screen-POP chemicals for each of the profiles range from 0 (hazard score lower than all chemicals in the reference set) to 100 (hazard score greater than all chemicals in the reference set).

Details on the reference set and the benchmarking method can be found in our previous publication (Reppas-Chrysovitsinos et al. 2017). In brief, the reference set consists of 148 well-characterized chemicals that are used to rank and contextualize the hazard profiles of chemicals with which we have less experience and information on a comparative scale.

The Screen-POP database was obtained by request from the authors of the original Screen-POP study (Arnot et al. 2012). Details on the initial compilation of each of the datasets can be found in these studies. The partition ratios and degradation half-lives for both the Screen-POP and reference databases were derived from EPI Suite, but in slightly different ways. The log *K*_AW_ and log *K*_OW_ values of the reference set were estimated by the HENRYWIN and KOWWIN modules of EPI Suite 4.11, respectively, and no further modifications were applied. EPI Suite 4.11 was also used to estimate degradation half-lives in air (t_1/2_air), water (t_1/2_water) and soil (t_1/2_soil) that are required as input for the OECD Tool for both the Screen-POP chemicals and the reference set chemicals. The log *K*_AW_ and log *K*_OW_ values in the Screen-POP database were selected by the Screen-POP authors, and are a combination of experimental values (when available in the EPI Suite database), log *K*_AW_ values calculated from estimated water solubility and vapor pressure (when HENRYWIN could not provide estimates), and HENRYWIN and KOWWIN estimates for any other case of log *K*_AW_ and, respectively, log *K*_OW_. Partition ratios with predicted values exceeding the extrema of the respective measured values that were used as EPI Suite’s training sets were considered to fall outside the model domain and were converted to the corresponding experimental extrema. Therefore, the log *K*_OW_ range is (−4, 10) and the log *K*_AW_ range is (−12, 3). The Tool outputs are known to become insensitive to changes of partition ratios outside the aforementioned range (Fenner et al. 2005) and therefore we incorporated this adjustment which also simplifies the comparison of the chemical spaces of the Screen-POP database and the reference set (− 10 < log *K*_AW_ < 2 and − 2 < log *K*_OW_ < 10).

Previous work with the Screen-POP database used an alternative method (Arnot et al. 2012) to obtain a more diverse set of degradation half-lives from the initial EPI Suite estimates. In this work, we used the default EPI Suite estimates for degradation half-lives of chemicals in both the reference and Screen-POP datasets. Although the half-life estimation method used in previous Screen-POP work has advantages, our goal in this study is to focus on the application of our method as presented earlier (Reppas-Chrysovitsinos et al. 2017) and further explore its efficacy to screen for planetary boundary threats without introducing new corrections and assumptions- which is also in line with the screening-level character of our method.

We removed from the Screen-POP database any chemical compound that is likely to be ionized within an environmentally relevant pH range (i.e. p*K*_a_ < 5 or p*K*_b_ > 8) because of uncertainties associated with their partitioning and their estimated *BAF*. For the estimation of p*K*_a_ and p*K*_b_ we employed the ACD Labs Percepta software (Advanced Chemistry Development Inc 2015). After this data curation step, a total of 8648 compounds remained from the original set of 12,619 in the Screen-POP database. Therefore, in the following lines the term “Screen-POP chemicals” is used to refer to these 8648 chemicals and not to the original set.

## Results and Discussion

A comparison of the structures contained in the Screen-POP and reference datasets is presented in Table 1. The molecular weight range of the Screen-POP chemicals is broader and more diverse than the reference set. The reference set has a higher percentage of halogenated compounds- which reflects its focus on known and well-characterized environmental contaminants- compared to the Screen-POP dataset which is representative of chemicals in commerce in OECD countries. Interestingly, differences in the number of halogen atoms between the two databases are not observed in a uniform manner; the occurrence of bromine and iodine atoms is similar in both sets while the percentage of chlorine is smaller and the percentage of compounds containing fluorine is larger in the Screen-POP database. It is also noteworthy that the reference set contains no organosilicons and organoborons since these compound groups were not represented among well-characterized environmental pollutants at the time the data for the reference set was collected.

**Table 1:** Structural descriptors of the Screen-POP and reference sets of chemicals

Molecular descriptors	Reference set (n = 148)	Screen-POP database (n = 8648)
Molecular weight (g/mol)	Min = 54, max = 545, median = 179, mean = 200	Min = 16, max = 1504, median = 214, mean = 267
Contains atoms of		
F	1.35% (2 compounds)	3.80% (328 compounds)
Cl	49.3% (73 compounds)	13.1% (1132 compounds)
Br	2.7% (4 compounds)	2.93% (253 compounds)
I	0.67% (1 compound)	0.30% (26 compounds)
N	22.2% (33 compounds)	36.3% (3316 compounds)
S	9.45% (14 compounds)	10.1% (873 compounds)
P	3.37% (5 compounds)	3.02% (261 compounds)
O	50.6% (75 compounds)	81.6% (7061 compounds)
Si	0% (0 compounds)	2.82% (244 compounds)
B	0% (0 compounds)	0.30% (26 compounds)

**Fig. 1:**
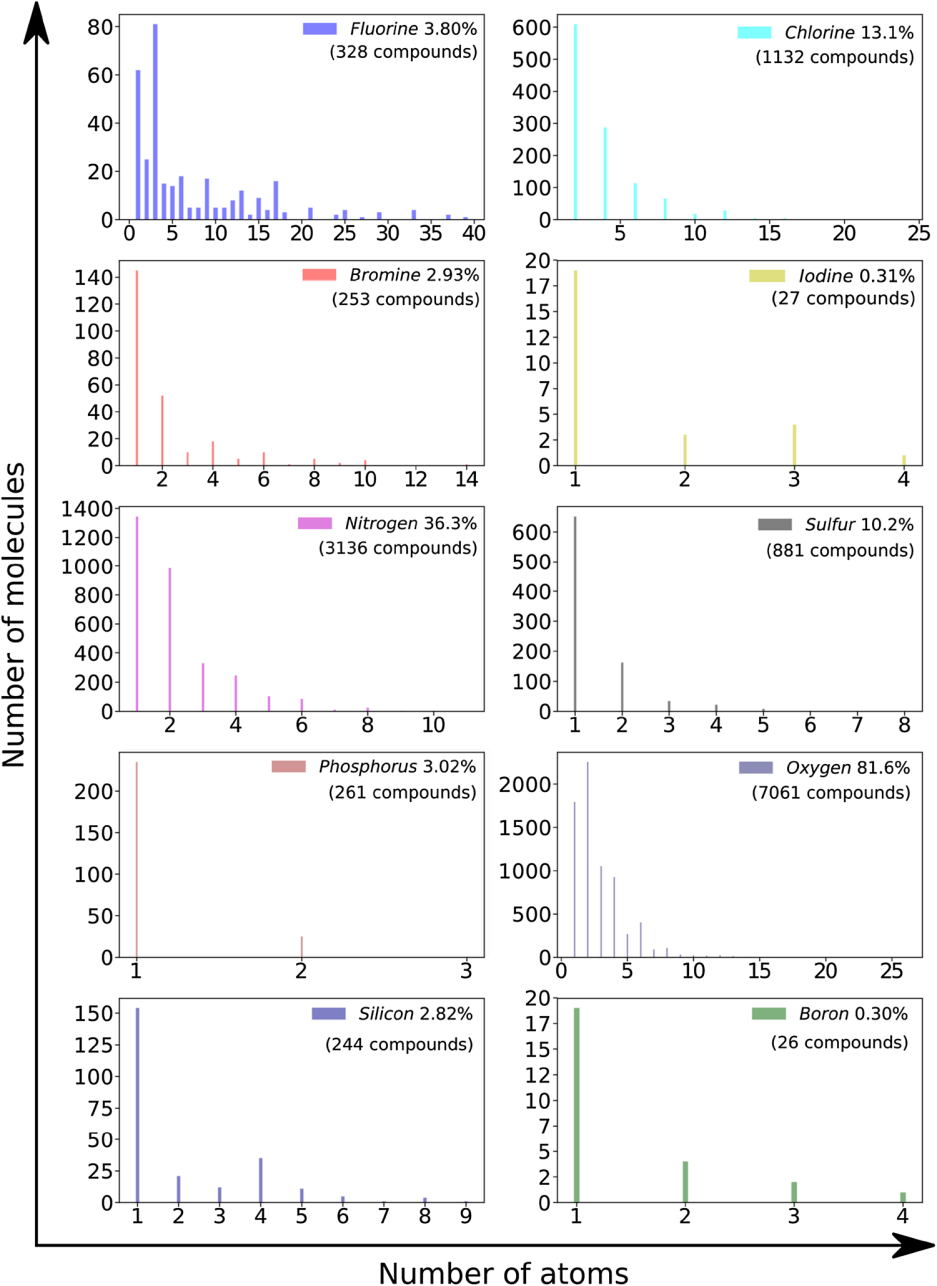
Frequency distribution of Screen-POP chemicals by the number of specific atoms in their structures

A more detailed analysis of the structural composition of the Screen-POP chemicals is presented in Fig. 1, which illustrates the frequency distribution of the number of atoms included in the structures of the Screen-POP chemicals for each of the ten elements found in Table 1. Most frequently the fluorinated compounds in the Screen-POP database have three atoms of fluorine in their structure, which is presumably a terminal –CF_3_ group. The second most common fluorination level is one fluorine atom and the third is two fluorine atoms. More than half of the 1132 chlorinated compounds contain two chlorine atoms with the second most frequent chlorination level being four chlorine atoms. The majority of the 253 brominated compounds, as well as the majority of the 27 iodinated chemicals include only one halogen atom in their structures. For fluorinated and iodinated chemicals an odd number of fluorine or iodine atoms is more frequent than an even number while for chlorinated and brominated chemicals an even number of halogens is more common.

**Fig. 2:**
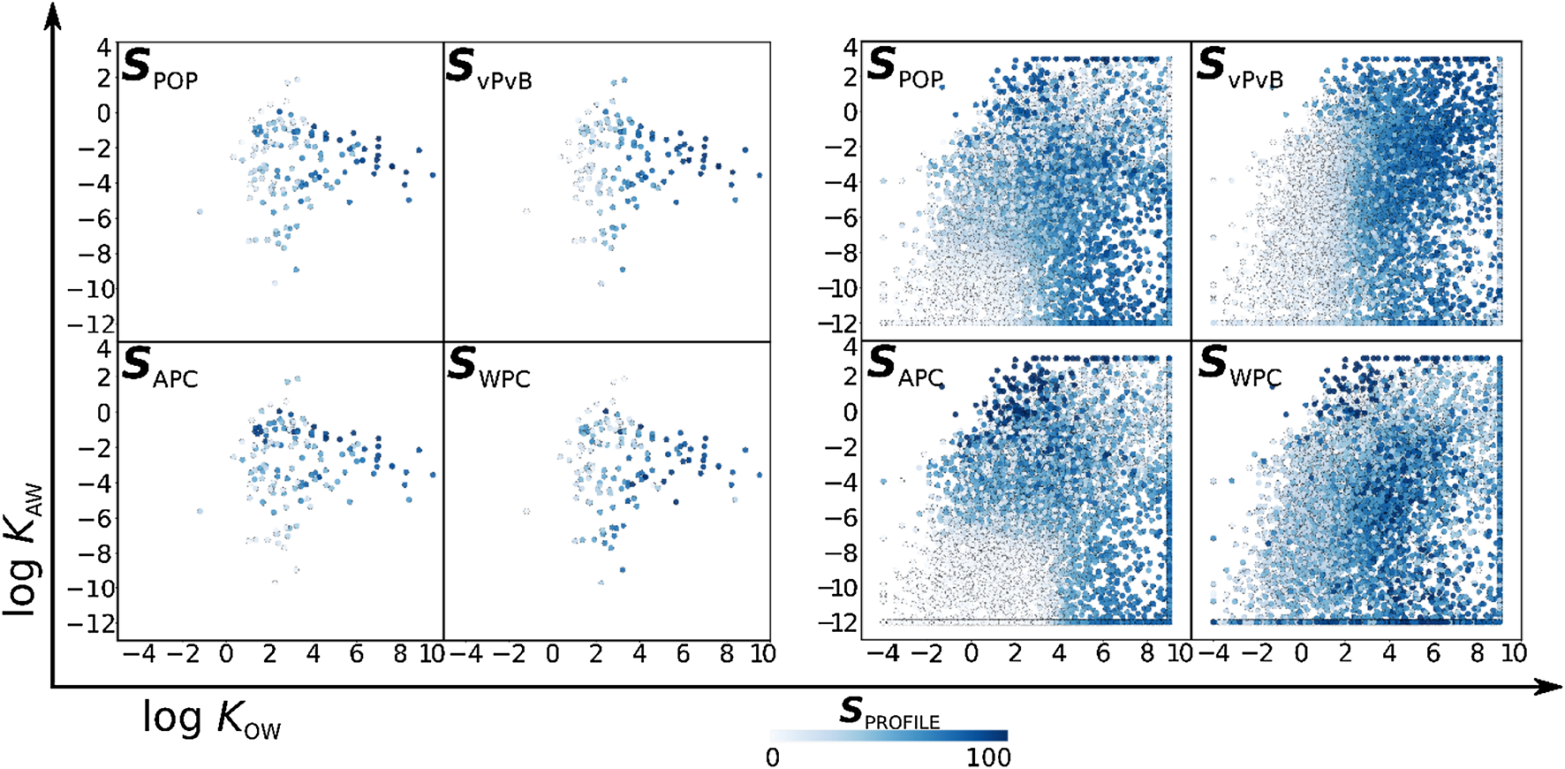
Chemical partitioning space showing the partition ratios between air, water and octanol of the reference set (left-hand side) and the Screen-POP chemicals (right-hand side). The relative influence of the partitioning properties on each of the scores is illustrated with higher scores depicted with darker color

Figure 2 depicts the three dimensional (log *K*_AW_, log *K*_OW_, ***S***_PROFILE_) chemical space of the reference set (left-hand side) and the Screen-POP database (right-hand side). In general, both databases contain hydrophobic (log *K*_OW_ > 6) and water-soluble (log *K*_AW_ < − 8) chemicals. The Screen-POP set includes some very volatile compounds (log *K*_AW_ > 2) that are not represented in the reference set.

**Fig. 3:**
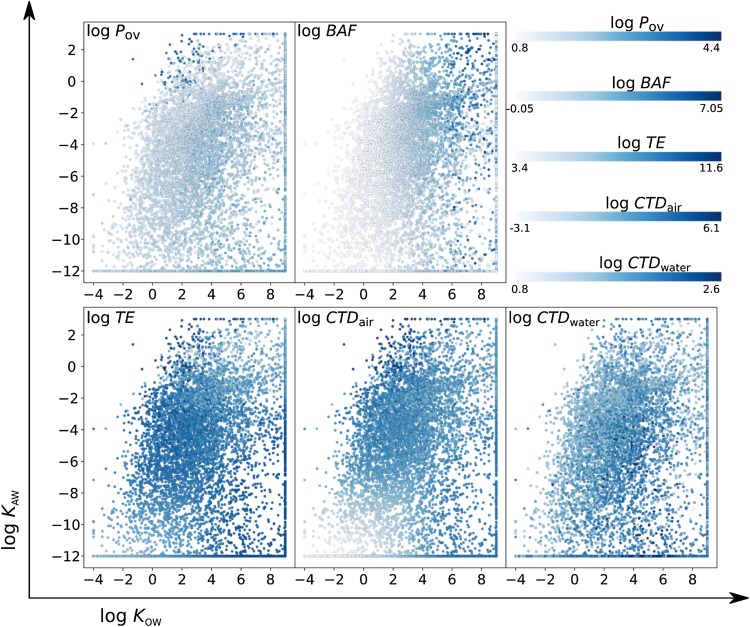
The relative influence of the partitioning properties (log K_AW_, log K_OW_) on each of the metrics (log P_OV,_ log BAF, log TE, log CTD_air_ and log CTD_water_) illustrated with higher values is depicted with darker color

Each of the two panels in Fig. 2 consists of four subplots that provide an overview of the relationship between partitioning properties and the distribution of scores for chemicals according to each of the four profiles. Given its size and diversity, the Screen-POP data serves as good basis to elucidate relationships between partitioning and hazard and to identify general trends. For example, in the case of the APC profile, the chemical space region occupied by highly water soluble chemicals (bottom left corner) has the lowest ***S***_APC_ values (light grey color) as highly water soluble chemicals are not likely to have high *CTD*_air_ values. Similarly—with the exception of some very persistent chemicals—volatile chemicals score low in the WPC profile. In the ***S***_vPvB_ panel, we observe that most of the changes in the score occur along the horizontal (*K*_OW_) axis, which reflects the influence of *K*_OW_ on *BAF* that is built into the BCFBAF model in EPI Suite, and also illustrated in Fig. 3.

Figure 3 consists of five three dimensional (log *K*_AW_, log *K*_OW_, hazard metric) subplots, each illustrating the relationship between chemical partitioning properties (log *K*_AW_, log *K*_OW_) and each of the five individual hazard metrics (log *P*_ov_, log *BAF*, log *TE*, log *CTD*_air_ and log *CTD*_water_) for the Screen-POP chemicals. In general, high persistence is associated with higher log *K*_OW_ values however, the most persistent Screen-POP chemicals are volatile and semi-volatile molecules (indicated with dark blue). Notably, these Screen-POP compounds score very high in every profile, as illustrated in Fig. 2. *BAF* is positively associated with log *K*_OW_, as expected by the BCFBAF model in EPI Suite. Highly water soluble chemicals (log *K*_AW_ < − 9 and log *K*_OW_ < 1) are not efficiently transported through the atmosphere in our model calculations and hydrophobic and volatile chemicals (log *K*_AW_ > 1 and log *K*_OW_ > 6) are not likely to be deposited and thus they have the lowest log *TE* values, in agreement with the definition of this metric. Volatile chemicals have high log *CTD*_air_ values and highly water soluble chemicals the lowest ones. In contrast to the log *TE* case, hydrophobic and volatile chemicals have high log *CTD*_air_ values for the same reason; they are likely to be transported without being deposited. Similarly, such chemicals- when emitted in water- favor partitioning to particles and are transported with them in the water. Apart from these chemicals, water-soluble molecules that favor the dissolved phase in water also have high log *CTD*_water_ values.

**Fig. 4:**
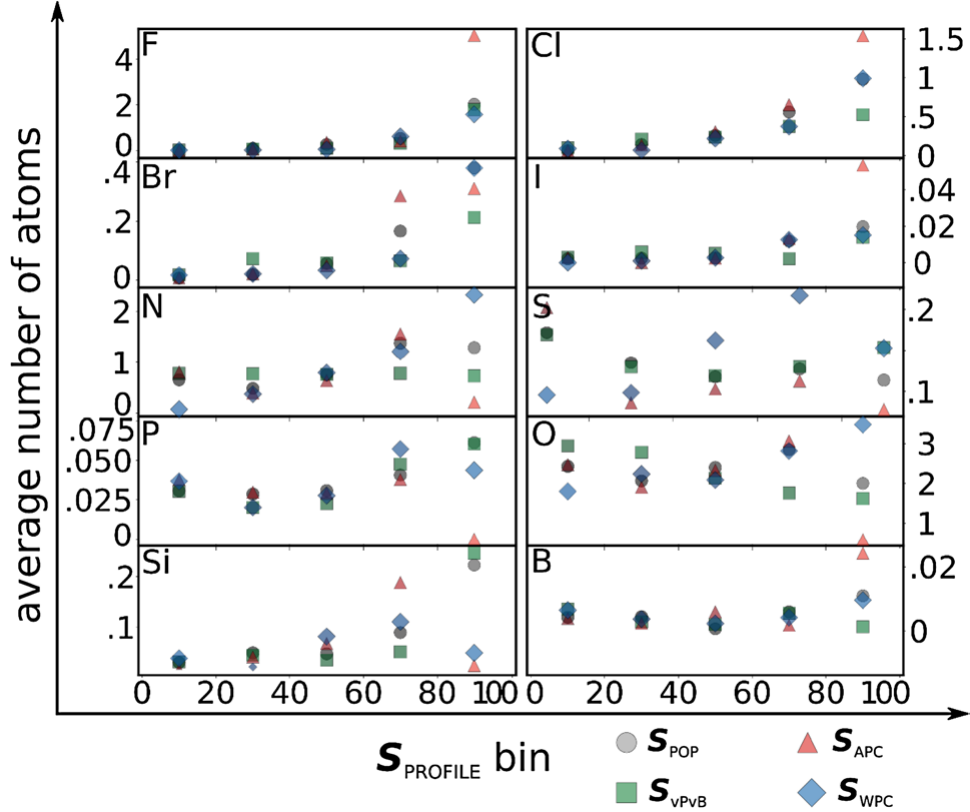
Relationship between the average number of a specific element in the chemical structure and the hazard score (***S***_PROFILE_). The elements considered are fluorine (F), chlorine (Cl), bromine (Br), iodine (I), nitrogen (N), sulfur (S), phosphorus (P), oxygen (O), silicon (Si), and boron (B)

Figure 4 illustrates the influence of the presence of different atoms in the structures of the Screen-POP molecules- represented by the average number of atoms of this element per structure- on their hazard scores. More specifically, we observe that the presence of halogens in the molecular structure is associated with higher scores in all profiles while the presence of oxygen in the molecule is associated with lower scores for all profiles except for WPC. The presence of nitrogen and sulfur in the molecular structure is associated with higher ***S***_WPC_ scores but lower ***S***_POP_, ***S***_vPvB_ and ***S***_APC_ scores. Phosphorus seems to be generally associated with lower scores in every profile, and the presence of silicon is associated with higher estimated log *K*_OW_ values and, therefore, higher *BAF* values and, consequently, higher ***S***_POP_ and ***S***_vPvB_ scores. Interestingly, organoboron Screen-POP compounds appear within the ***S***_APC_90 and ***S***_WPC_90 chemicals but not among the higher scoring Screen-POP chemicals in the “traditional” profiles.

**Fig. 5:**
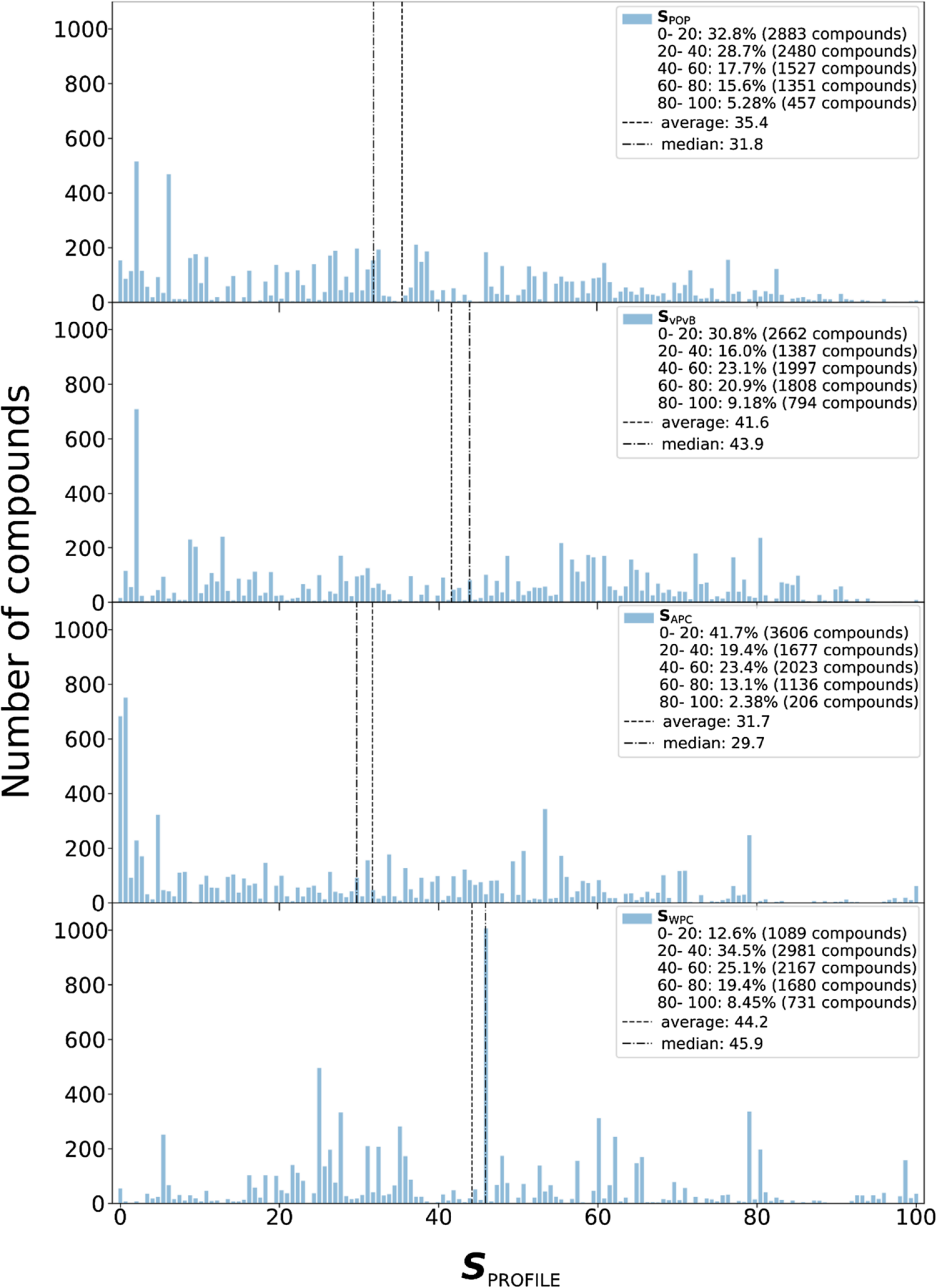
Frequency distribution of hazard scores as (from top to bottom) persistent organic pollutants (POP), very persistent and very bioaccumulative (vPvB), airborne persistent chemicals (APC) and waterborne persistent chemicals (WPC)

In general, the average score as well as the median of the scores for each hazard profile for the Screen-POP chemicals is below 50, as presented in Fig. 5. These scores do not have any regulatory or risk significance, but reference set chemicals that define the boundaries of the score can be used to provide comparative context. Chlordane, 1,2,3,4,7,8-hexachlorodibenzodioxin, chloropiricin and 2,3,7,8-tetrachlororodibenzodioxin are the reference set chemicals that mark the top 10% interval for ***S***_POP_, ***S***_vPvB_, ***S***_APC_ and ***S***_WPC_ respectively. We denote as ***S***_PROFILE_90 the Screen-POP chemicals that scored over 90, i.e. chemicals that scored over the aforementioned reference set chemicals in each of the respective profiles. Similarly, we denote as **S**_PROFILE_10 the Screen-POP chemicals that scored below 10. In total, 577 Screen-POP compounds scored over 90 in at least one of the four profiles, 80 Screen-POP chemicals did not score over 10 in any of the profiles, and six chemicals (sorbitol, inositol, isomaltitol, lactitol, polyvinyl alcohol and acarbose) scored 0 in all four profiles.

Four fluorinated chemicals exhibit such high estimated degradation half-lives in air that they scored 100 in every profile (Table 2) and seven more fluorinated chemicals scored over 90 in all four profiles (see Supporting Information). These eleven chemicals- the high scores of which are mostly attributable to very high estimated *P*_ov_ rather than their partitioning properties- indicate the potentially dominant role of extremely high persistence in profiling chemicals for high exposure hazard.

**Table 2:**
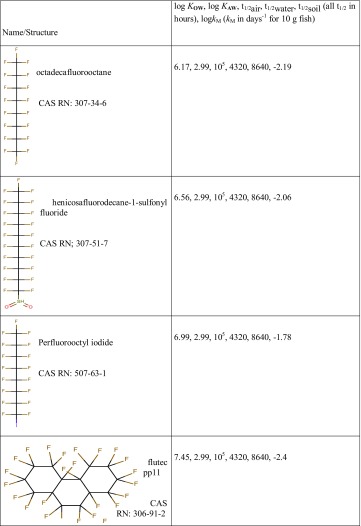
Chemicals selected from the Screen-POP database that scored 100 in all profiles (***S***_PROFILE_100)

A further exploration of the results illustrated in Fig. 4 showed that from the 97 chemicals that scored within ***S***_POP_90, 20 chemicals scored that high only in this profile. Similarly, 114 chemicals scored within ***S***_vPvB_90 and more than half of them (63) scored over 90 only in the vPvB profile. In the case of the APC profile, 54 out of the 155 chemicals that scored within ***S***_APC_90 did not score over 90 in any other profile and in the case of the WPC profile 299 of the 405 compounds that scored within ***S***_WPC_90 were below ***S***_PROFILE_90 in every other profile. Examples of chemicals that scored over 90 in only one of the profiles are provided in Table 3. Guaiene is a fragrance (The Good Scents Company 2017), chlorofluorocarbon 123 (National Center for Biotechnology Information 2017f) is a refrigerant used in low pressure refrigeration and heating, ventilation and air-conditioning systems which is scheduled for phasing out until 2040 under the Montreal protocol, and cyhalothrin (National Center for Biotechnology Information 2017a) is an incesticide. Monocarbinol terminated is an organosilicon used in food contact applications and not evaluated by an international body (Committee of Ministers-Council of Europe 1999). Monocarbinol is also included in a patent for a dye as well as being used as an additive to polymers that “offers better slip, antiblocking, mar resistance, surface smoothness, flexibility and hydrophobicity” (Siltech 2016).

**Table 3:**
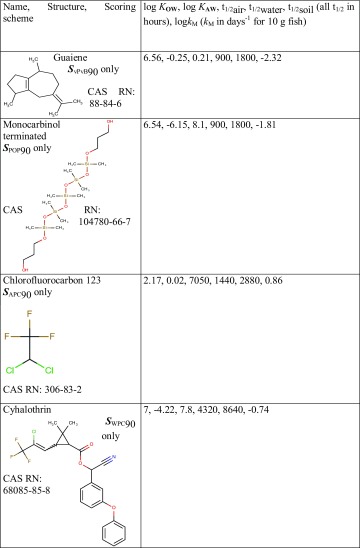
Examples of chemicals that scored over 90 (i.e. ***S***_PROFILE_90) in only one of the profiles

Although the exposure- based hazard profiles we used in this study are independent from each other, there are also conceptual and practical overlaps among them. We can combine the scores into schemes that reveal the most relevant profiles for potential regulatory action from the perspective of the spatial coverage of their exposure potential. Chemicals that fit either of the APC or WPC profiles but not the POP profile are expected to be mostly relevant for regulatory action at a supranational-to-global scale. Similarly, chemicals that fit the POP profile but not the vPvB one are expected to be mostly relevant for regulatory action at a national-to-supranational scale (far-field exposure) while chemicals that fit the vPvB profile are of particular interest predominantly on a national scale (near-field exposure). Table 4 presents examples of such Screen-POP chemicals. Disulfide, bis(3,3,4,4,5,5-hexafluorohexyl) is a commenced premanufacture notice substance under the US toxic substances control act (TSCA) along with the whole class of bis(gamma-omega-perfluoro-C6-20-alkyl) disulfides (National Center for Biotechnology Information 2017c), 2-phenyl-1,1-bis(1h-indol-1-yl)ethane is a fragrance (European Chemicals Agency 2017) and pentabromotoluene is mainly used as a flame retardant (National Center for Biotechnology Information 2017e). Tris((3-ethyl-3-oxetanyl)methyl) phosphite is an additive found in an inventory list of substances used for the manufacture of surface coatings intended to come into contact with foodstuffs as a substance not fully evaluated by an international body (Committee of Ministers-Council of Europe 1996). In total, 114 chemicals scored within ***S***_vPvB_90, 46 chemicals scored within ***S***_POP_90 and not ***S***_vPvB_90, 118 chemicals scored within ***S***_APC_90 and not ***S***_POP_90, and 363 chemicals scored within ***S***_WPC_90 and not ***S***_POP_90. Lists of these Screen-POP chemical groups are available in Supporting Information.

**Table 4:**
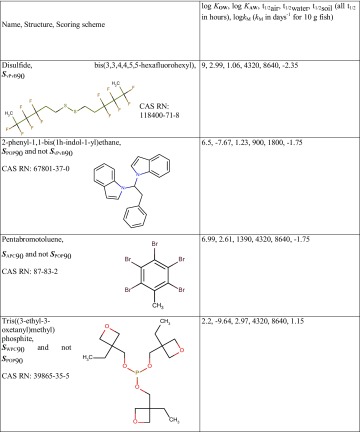
Examples of chemicals ordered according to a scoring scheme for the spatial coverage of the profile that they fit the best (***S***_PROFILE_ ≥ 90)

The Screen-POP database consists of chemicals that were reportedly produced in the OECD countries as of the year 2012. The chemical production of the OECD countries is estimated to account for at least half of the global chemical production volume (Sigman et al. 2001). Given the size and diversity of the Screen-POP database, our goal was to screen this set of chemicals for potential planetary boundary threats, POPs and vPvBs. We successfully identified high scoring Screen-POP chemicals that have a high potential to be POP, vPvB, APC and/or WPC candidates and we further proposed a scoring scheme that can be used to disentangle the complementary nature of these four profiles by focusing on the spatial range of their exposure potential. However, this approach to identify chemicals of concern, and especially potential planetary boundary threats, does not regard the socioeconomic modes of transfer of pollutants as exposure pathways (MacLeod et al. 2014; Ng and Goetz 2016).

The majority of the Screen-POP chemicals examined in this study scored low relative to the reference set in all profiles as the Screen-POP database is not contaminant-focused. However, scores should not be interpreted as a direct proxy for “risk” because possible effects are not explicitly considered in this assessment. Moreover, the actual emissions of these chemicals are necessary to estimate their exposure and also, occasionally, there are significant differences between the actual and the estimated physicochemical properties which define these scores. More specifically, EPI Suite is known to underestimate degradation half-lives of persistent chemicals (Gouin et al. 2004) and the physicochemical properties of some chemical classes, such as organosilicons and organoborons, which are underrepresented in EPI Suite’s training sets, are subject to extremely high uncertainty. Various Screen-POP chemicals are not expected to fall equally well within the applicability domain of all the different EPI Suite QSARs (HENRYWIN, KOWWIN, BCFBAF, AOPWIN and BIOWIN) used in this assessment and, therefore, chemicals that scored high should be assigned high priority for experimental estimation of their physicochemical properties.

In addition to chlorofluorocarbon 123, our method assigned a high ***S***_APC_ score to decabromobiphenyl (National Center for Biotechnology Information 2017b) and dichloromethane (National Center for Biotechnology Information 2017d). A high ***S***_APC_ score for decabromobiphenyl is unexpected as this is a chemical with low vapor pressure and therefore its presence in air can only be attributed to its sorption to air particles. Although chemicals that are highly sorbed to atmospheric particles may be transported in air if they are emitted to air, it is questionable whether the atmospheric half-lives of aerosol particles are sufficient to render such low-volatility chemicals of concern as APCs. Therefore, we identify the need to further explore the relationship between partitioning properties and scores and specifically examine the appropriateness of the ***S***_APC_ score to capture potential APCs. On the other hand, dichloromethane, which is present both in the reference set and the Screen-POP database, was recently identified as an ozone depleting chemical (Hossaini et al. 2017). This finding illustrates the utility of our ranking method and highlights the relative advantages of using a reference set rather than adopting “bright line” criteria in prioritization assessments.

Screening-level studies are highly dependent on data availability and data quality. The in silico tools and methodologies developed thus far have enabled estimation of property data and provided a rigorous framework to identify and prioritize organic chemicals for unacceptable environmental behavior. Therefore, the main limitations of such studies are on the data availability side, both in terms of measurements of property data of diverse substances to train QSPR models, and representativeness of the databases of chemicals used in the screening exercise. The Screen-POP database is large, diverse, focused on chemicals that were actually produced at least up to 2011, and- by design- covers an important part of the global chemical production. However, this database is a snapshot. Since this dataset was compiled, several new individual chemicals have been introduced in the market while information on the chemical production of non-OECD countries is scarce. Therefore, field studies and analytical approaches are necessary to identify new contaminants that would otherwise remain undiscovered (Sobek et al. 2016; Blum et al. 2018).

Future studies building on our approach to screen chemicals for high exposure hazard potential should aim to expand the reference set currently used to better account for more diverse chemical databases, expand the methodology to include ionic and ionizing chemicals, improve on the EPI Suite-estimated degradation half-lives, and further investigate the influence of the partitioning properties on the profile scores.

## Electronic supplementary material

Below is the link to the electronic supplementary material.

Supplementary material 1 (XLSX 15556 KB)
